# Why Do Herbivorous Mites Suppress Plant Defenses?

**DOI:** 10.3389/fpls.2018.01057

**Published:** 2018-07-30

**Authors:** C. Joséphine H. Blaazer, Ernesto A. Villacis-Perez, Rachid Chafi, Thomas Van Leeuwen, Merijn R. Kant, Bernardus C. J. Schimmel

**Affiliations:** ^1^Department of Evolutionary and Population Biology, Institute for Biodiversity and Ecosystem Dynamics, University of Amsterdam, Amsterdam, Netherlands; ^2^Department of Plants and Crops, Faculty of Bioscience Engineering, Ghent University, Ghent, Belgium

**Keywords:** defense suppression, host plant manipulation, resistance, *Tetranychus*, effectors, jasmonate, herbivore, buffering trait

## Abstract

Plants have evolved numerous defensive traits that enable them to resist herbivores. In turn, this resistance has selected for herbivores that can cope with defenses by either avoiding, resisting or suppressing them. Several species of herbivorous mites, such as the spider mites *Tetranychus urticae* and *Tetranychus evansi*, were found to maximize their performance by suppressing inducible plant defenses. At first glimpse it seems obvious why such a trait will be favored by natural selection. However, defense suppression appeared to readily backfire since mites that do so also make their host plant more suitable for competitors and their offspring more attractive for natural enemies. This, together with the fact that spider mites are infamous for their ability to resist (plant) toxins directly, justifies the question as to why traits that allow mites to suppress defenses nonetheless seem to be relatively common? We argue that this trait may facilitate generalist herbivores, like *T. urticae*, to colonize new host species. While specific detoxification mechanisms may, on average, be suitable only on a narrow range of similar hosts, defense suppression may be more broadly effective, provided it operates by targeting conserved plant signaling components. If so, resistance and suppression may be under frequency-dependent selection and be maintained as a polymorphism in generalist mite populations. In that case, the defense suppression trait may be under rapid positive selection in subpopulations that have recently colonized a new host but may erode in relatively isolated populations in which host-specific detoxification mechanisms emerge. Although there is empirical evidence to support these scenarios, it contradicts the observation that several of the mite species found to suppress plant defenses actually are relatively specialized. We argue that in these cases buffering traits may enable such mites to mitigate the negative side effects of suppression in natural communities and thus shield this trait from natural selection.

## Introduction

Among the diverse organisms that parasitize plants are numerous species of mites (Arachnida: Acari). With a body size of usually less than a millimeter, these mites are among the smallest herbivores. They feed by piercing an epidermal or mesophyll cell with their stylet-like mouthparts, after which they suck up the cellular contents (Helle and Sabelis, 1985; Lindquist et al., 1996; Bensoussan et al., 2016). Despite their relatively limited per capita consumption, herbivorous mites are a pest on nearly every agriculturally or horticulturally important plant species, causing massive economic losses worldwide (Helle and Sabelis, 1985; Lindquist et al., 1996; Van Leeuwen et al., 2015). That is because herbivorous mites generally have high fecundity, a short developmental time and a female-biased offspring ratio, which, among others, allows them to build up populations large enough to destroy entire plants within just a few weeks (Helle and Sabelis, 1985; Lindquist et al., 1996).

**FIGURE 1:**
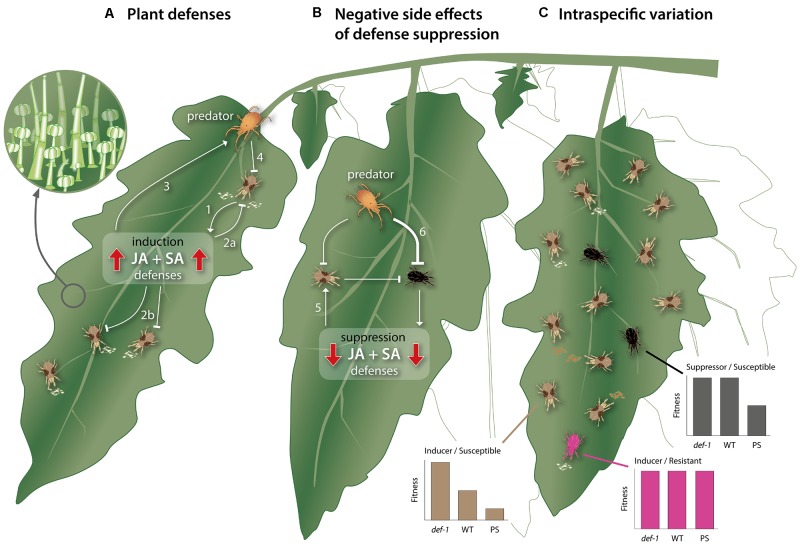
Schematic, simplified overview of the direct and indirect interactions between cultivated tomato plants, herbivorous spider mites, and carnivorous predatory mites. **(A)** Mite herbivory induces direct and indirect plant defenses that hamper mite performance. While feeding, *Tetranychus urticae* triggers the accumulation of the phytohormones jasmonic acid (JA) and salicylic acid (SA), which, in turn, promotes the production of (volatile) metabolites and proteins (arrow 1), aimed at harming or deterring the mite (inhibition line 2a). Such plant-produced metabolites and proteins may similarly affect mites that simultaneously or subsequently feed from the same or from not previously attacked, systemic tissues (inhibition line 2b). Additionally, they may mediate the attraction and arrestment of the attacker’s natural enemies (arrow 3), thereby facilitating predation of the herbivore (inhibition line 4). The areal surface of tomato plants contains glandular trichomes (inset), which not only represent a physical barrier for mites, but also produce large amounts of defense-associated (volatile) metabolites and proteins. **(B)** In natural communities, herbivorous mites that suppress plant defenses may suffer from negative side effects associated with this trait. That is, by suppressing defenses, the host becomes a better food source, including for competitors, which may therefore be promoted on a shared leaf (arrow 5). Furthermore, defense-suppressing mites themselves may become a better food source for their natural enemies, i.e., better than defense-inducing mites (inhibition line 6). **(C)** For more polyphagous mite species, intraspecific variation in traits related to mite–plant interactions exists within natural populations. Three *T. urticae* genotypes have, for instance, been described: (i) Mites that induce defenses on wild type (WT) tomato and that are susceptible to these defenses (brown mites). Consequently, these mites have a low fitness on WT plants, a much higher fitness on JA-impaired mutants (*def-1*) and an extremely low performance on transgenic 35S::prosystemin (PS) plants that constitutively display JA defense responses. (ii) Mites that are susceptible to tomato defenses but nevertheless maintain a high fitness because they suppress these defenses (black mites). These mites perform as well on WT as on *def-1*, but have a lower fitness on PS, presumably because they cannot suppress the extraordinarily strong defenses of PS plants. Mites from this genotype appear to be relatively rare as compared to the defense-inducing/susceptible ones. (iii) Mites that induce defenses but that are metabolically resistant to plant-produced defensive metabolites or proteins and, therefore, have an equally high fitness on WT, *def-1* and PS (pink mites). This genotype appears to be rare as well. Note that mites are not drawn to scale and are distinctively colored for illustrative purposes only. In nature, *T. urticae* from the three described genotypes are, by eye, morphologically indistinguishable.

In order to protect themselves from mites and other phytophagous organisms, plants have evolved a plethora of defensive traits. A subset of these traits is aimed at deterring, inhibiting or killing the parasite via mechanisms that range from physical obstruction to the production of (volatile) metabolites or proteins that either directly harm the attacker, e.g., due to their toxic or antinutritional properties, or that do so indirectly via facilitating the recruitment of the attacker’s natural enemies (**Figure 1A**) (Jones and Dangl, 2006; Heil, 2008; Mithöfer and Boland, 2012; Schuman and Baldwin, 2016). A major source of resistance to small arthropod herbivores, including mites, are the glandular trichomes, as these represent physical barriers that also produce, store and/or exude large amounts of (volatile) defensive metabolites and proteins (Glas et al., 2012). Many defensive traits, however, are more specific in the sense that they: (a) are most effective against a relatively narrow range of attackers, and; (b) are confined to a single plant species, -family, -order or -clade (Mithöfer and Boland, 2012; Couto and Zipfel, 2016; Schuman and Baldwin, 2016). Furthermore, in many cases the amounts of defensive metabolites and proteins are low or absent under unstressed conditions but increase considerably upon attack. Probably, inducible defenses have evolved to save resources and/or to limit autotoxic effects (Mithöfer and Boland, 2012; Couto and Zipfel, 2016; Schuman and Baldwin, 2016). Such inducibility requires a rapid and robust signaling machinery to activate the appropriate defenses in a timely manner, which starts with detection of the attacker.

How plants recognize mite-feeding or the extent to which they can tell mites apart from other herbivores, is yet unknown. Plants are thought to recognize their attacker based on the perception of two classes of molecules: The first one being damage-associated molecular patterns (DAMPs), which are plant-derived and modified or dislocated as a consequence of wounding (Heil and Land, 2014; Gust et al., 2017). Spider mites seem to avoid causing unnecessary damage and, hence, the release of DAMPs, by inserting their stylet in between epidermal cells or via open stomata to reach the mesophyll (Bensoussan et al., 2016). The second one being microbe- or herbivore-associated molecular patterns (MAMPs or HAMPs, respectively), which can be attacker-derived, plant-derived (but often modified by the attacker) or conjugates of the two (Boller and Felix, 2009; Acevedo et al., 2015). DAMP-, MAMP-, and presumably also HAMP-recognition is mediated by pattern-recognition receptor (PRR) proteins (Couto and Zipfel, 2016). Such recognition activates an intracellular signaling cascade that, within minutes, results in the induction of defenses (Couto and Zipfel, 2016). This entire process is known as pattern-triggered immunity (PTI). Whereas PTI has been well established for plants attacked by diverse microbial phytopathogens and is expected to function in plant–herbivore interactions as well, experimental evidence for the latter hypothesis is still scarce and often indirect. For instance, while several HAMPs have been characterized at the molecular level, no matching PRR has been identified (Acevedo et al., 2015; Schmelz, 2015). Likewise, some plant PRRs have been implicated in plant resistance to herbivores (Gilardoni et al., 2011; Cheng et al., 2013; Gouhier-Darimont et al., 2013; Liu et al., 2015; Hu et al., 2018) but the matching herbivory-derived ligands remain elusive. Hence, while it is likely that they exist, mite-derived HAMPs and cognate plant PRRs have not been identified yet.

The PTI signaling cascade critically depends on the action of several phytohormones, the most important ones are jasmonic acid (JA) and salicylic acid (SA) (Pieterse et al., 2012; Couto and Zipfel, 2016). Generally, JA is required to mount effective defense responses against herbivores as well as microbial pathogens with a necrotrophic life style (Pieterse et al., 2012). By contrast, resistance against biotrophic microbial pathogens depends on SA (Glazebrook, 2005; Pieterse et al., 2012). Curiously, some mites, including the extremely polyphagous cosmopolitan pest species *Tetranychus urticae*, simultaneously induce JA and SA-regulated defenses (**Figure 1A**) (Kant et al., 2004; Zhurov et al., 2014; Alba et al., 2015; Schimmel et al., 2017a,b), although plant resistance to these mites predominantly depends on JA (Kant et al., 2008; Zhurov et al., 2014; Villarroel et al., 2016). Signaling components of the JA and SA pathways can interact with each other, synergistically or antagonistically, but can also interact with signaling components of growth-regulating hormonal pathways (Pieterse et al., 2012). Hormonal crosstalk is thought to adaptively tailor defense responses to different enemies as well as to minimize wasting resources on unnecessary defenses (Thaler et al., 2012). Finally, many of the defense responses activated during PTI are induced not only in the attacked tissue but also in non-attacked, systemic tissues (Fu and Dong, 2013; Schuman and Baldwin, 2016). Herbivorous mites, too, induce defense responses in systemic leaves (Sarmento et al., 2011a), which may increase the resistance of these tissues when attacked later on (Agut et al., 2016).

Pattern-triggered immunity confers resistance to the majority of all plant parasites, meaning to those that induce defenses and are susceptible to them. Many phytophagous organisms, however, have acquired traits that enable them to overcome plant defenses (Kant et al., 2015). These traits can roughly be divided into three categories: (1) avoidance, (2) metabolic resistance, and (3) suppression, i.e., host plant manipulation. The avoidance of (induced) plant defenses entails a behavioral strategy that has been observed for diverse arthropod herbivores (Dussourd, 2017) at spatial resolutions ranging from between individual plants (Kessler et al., 2004) to within a single leaflet (Shroff et al., 2008). Metabolic resistance to plant defenses can arise from target-site insensitivity or from detoxification mechanisms that may include metabolite modification, degradation and/or secretion (Despres et al., 2007; Heckel, 2014). Defense suppression is achieved via sabotage of the host plant’s molecular machinery. To do so, plant-feeding organisms have evolved specialized molecules which they secrete into or onto their host and which interfere in various ways with the host’s ability to defend itself (Hogenhout and Bos, 2011; Kant et al., 2015; Khan et al., 2018). Such molecules are referred to as “effectors” or “virulence factors,” but it is important to point out that effectors that suppress defenses in one host plant may elicit defenses in another (in the latter case they are also referred to as “avirulence factors”) (Hogenhout et al., 2009). Here we will use the term “effector” in the context of host-plant defense suppression. Finally, it is of note that symbioses with microorganisms, or alternatively horizontal gene transfer events from microbes, may underlie a herbivore’s ability to overcome plant defenses (Douglas, 2015; Wybouw et al., 2016).

Among herbivorous arthropods, suppression of defenses has been observed in several insect species (Stahl et al., 2018), three spider mites species (*T. urticae*: Kant et al., 2008; *T. evansi*: Sarmento et al., 2011a; *T. ludeni*: Godinho et al., 2016) and an eriophyoid mite species (*Aculops lycopersici:* Glas et al., 2014). Research on defense suppression by arthropods has almost exclusively focused on the source of suppression (i.e., effectors) and the effect of suppression on plant physiology (Stahl et al., 2018). Defense suppression has obvious benefits for herbivores as the down-regulation of defenses coincides with an increase in herbivore fitness (Kant et al., 2008; Sarmento et al., 2011a; Alba et al., 2015; Schimmel et al., 2017a). This observation comes mostly from laboratory experiments lacking the natural ecological context. Yet, for understanding which factors drive the emergence, persistence, and disappearance of this trait this context may be crucial. For example, defense suppression appeared not only to benefit the herbivore doing it but also its competitors residing on the same leaf (Kant et al., 2008; Sarmento et al., 2011b; Alba et al., 2015) and may underlie patterns of mite species-succession observed in the field (Glas et al., 2014). In addition, defense suppression may promote predation (**Figure 1B**), given that the predatory mite *Phytoseiulus longipes* consumed more spider mite eggs that had been produced on defense-suppressed plants than eggs produced on plants with induced defenses (Ataide et al., 2016). This suggests that plant defensive substances, produced in response to herbivory, are transferred to the mite’s eggs and may hamper predators, unless defenses are suppressed. In nature, mites commonly live in close proximity to other herbivorous- and predatory mites, i.e., on the same plant or even on the same leaf (De Moraes and Lima, 1983; Rosa et al., 2005; Ferragut et al., 2013; Glas et al., 2014). If so, then how do defense-suppressing mites control the apparent ecological costs of suppression? This question becomes even more puzzling when considering that species like *T. urticae* possess an extraordinary number of genes associated with metabolic resistance (Grbić et al., 2011; Dermauw et al., 2013) and, hence, may not need to suppress defenses in the first place. By means of this review, we propose scenarios that may explain why defense suppression seems to be a relatively common trait among specialist as well as generalist plant-feeding mites.

We will first present the mechanistic background of defense suppression by herbivorous mites, including how to experimentally tell suppression apart from induction or from stealth feeding. Then, we will explore the eco-evolutionary scenario’s that may favor this trait. Finally, we will outline which traits may enable herbivorous mites to counteract the negative side effects of defense suppression that can occur when living in natural communities. We will focus on the direct and indirect interactions between cultivated tomato (*Solanum lycopersicum*), the generalist two-spotted spider mite (*T. urticae*), and the specialist tomato red spider mite (*T*. *evansi*), because these three species have become a model for addressing mechanistic or ecological questions on the costs and benefits of defense induction versus suppression by arthropod herbivores.

## Mechanistic Background of Plant Defense Suppression by Mites

The ability to suppress plant defenses is a trait that allows a phytophagous organism to lower the magnitude of a defensive process, either constitutive or induced, such that it gains a reproductive advantage. Although this definition could include behavioral sabotage such as vein-cutting (Dussourd, 2017), we will focus here on the suppression of molecular processes. The definition also excludes stealth feeding (Walling, 2008), because this does not affect the defensive process as such. It is important to realize that suppression does not need to be absolute, i.e., down to- or below levels of non-attacked plants, as it can already be effective when defenses are down-regulated to intermediate levels (Glas et al., 2014; Alba et al., 2015). In our experience, such absolute suppression is rare. Defense-suppressors rather reduce the extent to which a subset of defenses are induced (Glas et al., 2014; Alba et al., 2015). For example, when compared to non-infested controls, an infestation with defense-suppressing *T. urticae* or *T. evansi* typically results in the increased accumulation of JA and SA, as well as in the increased expression of defense-associated genes, yet the magnitude of these defense responses is very small when compared to an infestation with non-adapted *T. urticae* (Alba et al., 2015; Schimmel et al., 2017a,b). These properties make it challenging to experimentally tell suppression apart from induction as well as from stealth feeding. However, there are three selection criteria that, together, enable researchers to identify defense-suppressors.

The first of these criteria is that defense-suppressing mites should have a similar fitness on wild-type (WT) plants versus on defense-deficient mutants (**Figure 1C**). That is because suppression renders WT plants phenotypically equivalent to such mutants in terms of their susceptibility to herbivores. Indeed, whereas non-adapted *T. urticae* performed much better on the JA-biosynthesis mutant *defenseless-1* (*def-1*) than on WT tomato (Li et al., 2002), defense-suppressing *T. urticae* and *T. evansi* mites performed just as well on WT as on *def-1* plants (Kant et al., 2008; Alba et al., 2015). Since defense-resistant mites will also have an equally high fitness on WT and defense-deficient mutants, this assay can be expanded with a set of hyper-defended plants, such as transgenic *35S::prosystemin* (PS) plants, to further discriminate the suppressor mites from the defense-resistant ones (Kant et al., 2008). The idea behind this is that suppressors can no longer suppress the extraordinarily strong defenses of PS plants, while resistant mites remain unaffected by them (**Figure 1C**).

The second criterion is that, on a shared host, defense-suppressing mites should be able to facilitate conspecific and/or heterospecific mites, including non-adapted ones. The reasoning behind this is threefold: (1) Plants attacked by suppressor mites are a better food source than plants attacked by defense-inducing mites. This will translate into a higher herbivore fitness on the former. (2) Suppression is most likely not free of costs for mites, i.e., it requires resources/energy, thus also suppressors will benefit when defenses are already suppressed by others. (3) Mites that have adapted to plant defenses by not inducing them (avoidance) or by evolving insensitivity (metabolic resistance) will not pass this test, as they are unable to facilitate other mites. Accordingly, compared with their respective controls, non-adapted *T. urticae* had a higher reproductive performance when their tomato host was either previously or simultaneously infested with defense-suppressing *T. urticae* (Kant et al., 2008; Alba et al., 2015) or *T. evansi* (Sarmento et al., 2011b; Alba et al., 2015). Similar experiments have identified *T. ludeni* (Godinho et al., 2016) and *A. lycopersici* (Glas et al., 2014) as defense-suppressors. Despite the reported success of these co-infestation assays, they may also deliver variable results because the outcome strongly depends on the infestation conditions, such as timing of the infestations and the number of mites used (de Oliveira et al., 2016; Schimmel et al., 2017a,b). In addition, these co-infestation assays cannot discriminate between effects due to induced/suppressed defenses on the one hand and, for example, effects on plant resources on the other.

Whereas the first two criteria are bioassay-based and, thus, have mite performance as readout, the third criterion is based on a molecular assay and aimed to assess the impact of an alleged suppressor on an induced defense via an ask-the-plant approach. In practice this means that defense-suppressing mites should be able to suppress defenses that are induced by non-adapted mites or, in principle, by any other type of induction. The magnitude of defenses in plants that were infested with suppressor mites during or after the induction treatment should be lower than in plants that only received the induction treatment. For example, expression levels of defense-associated genes were significantly lower in tomato leaflets simultaneously infested with defense-suppressing *T. evansi* and defense-inducing *T. urticae* than in leaflets solely infested with defense-inducing *T. urticae* (Alba et al., 2015), even though the mite density was two-fold higher on the dual-infested leaves. This assay should be combined with one or both of the other methods as statistically significant down-regulation of defenses is by itself not proof for a biologically relevant effect. Finally, this assay may overlook relatively weak suppressors or suppressors with a primarily local effect.

**FIGURE 2:**
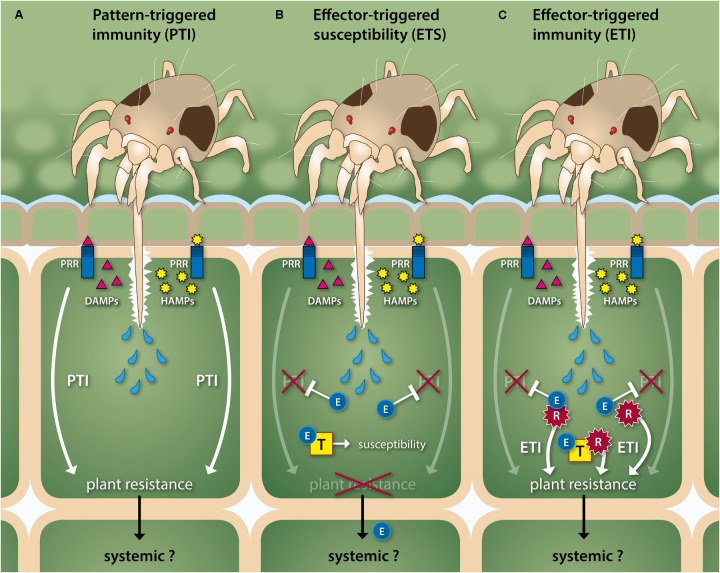
Schematic, simplified overview of the *in planta* molecular interplay between herbivorous spider mites and their host. **(A)** Spider mites use their stylet-shaped mouthparts to retrieve the contents of mesophyll cells, which may trigger the activation of plant defense responses that render the plant resistant. Mites pierce mesophyll cells and inject them with saliva prior to ingestion of their contents. This may cause the release of damage- and/or herbivore-associated molecular patterns (DAMPs and HAMPs, respectively) that are recognized by plant pattern-recognition receptors (PRRs) and which leads to pattern-triggered immunity (PTI). Spider mites seem to minimize the release of DAMPs by inserting their stylet via open stomata (not shown) or in between epidermal cells to reach the mesophyll. **(B)** Mites may interfere with PTI and other host processes by injecting salivary effector molecules (E) that target and interact with various plant proteins (T) to inhibit or to exploit their function and, thereby, render the plant susceptible. This process is termed effector-triggered susceptibility (ETS). **(C)** Plants have evolved receptor proteins (R) that detect effectors directly or indirectly and subsequently restore PTI responses plus induce additional defenses that altogether render the plant resistant again. This process is referred to as effector-triggered immunity (ETI). Herbivory by mites likely induces PTI- or ETI-associated defense responses beyond the attacked cell, i.e., also in non-attacked tissues, but the spatiotemporal dynamics of such systemic responses are not fully understood. Likewise, mites are thought to manipulate their host plant beyond the attacked cell, for instance via the intracellular transport of effectors. Note that mite-HAMP and plant-PRR pairs have not been identified yet. Mites, plant cells and their (secreted) components are not drawn to scale.

How suppression of plant defenses by mites, or by herbivorous arthropods in general, works at the molecular level is still poorly understood. Suppression by *T. urticae*, *T. evansi*, and *A. lycopersici* was found to act downstream from phytohormone accumulation and independently of JA–SA crosstalk (Glas et al., 2014; Alba et al., 2015). While feeding, mites secrete saliva, which contains effector proteins that sabotage the host’s defenses, resulting in effector-triggered susceptibility (**Figures 2A,B**) (Jonckheere et al., 2016; Villarroel et al., 2016). Combined genomic and transcriptomic analyses have revealed that spider mites are likely capable of producing and secreting several hundreds of salivary proteins (Jonckheere et al., 2016; Villarroel et al., 2016). Further proteomic analyses of salivary secretions collected using an artificial diet system have thus far identified 95 proteins from *T. urticae*’s saliva (Jonckheere et al., 2016). It remains unknown, though, how many of the (putative) salivary proteins actually interfere with the host’s defenses. Firstly, because the sequence identity of effectors is usually very species-specific thus hampering *in silico* identification (Arnold et al., 2009; Lo Presti et al., 2015). Secondly, effectors not necessarily target plant defenses to trigger host susceptibility (Van Schie and Takken, 2014; Macho, 2016). Thirdly, salivary proteins may have effector-unrelated functions. For example, several mite salivary proteins were predicted to be carbohydrate or protein catabolic enzymes, suggesting a role in the degradation of plant material, possibly prior to ingestion (Jonckheere et al., 2016). Lastly, salivary proteins may be multifunctional. Salivary proteases of insects, for instance, may serve to (pre)digest proteins as food but may additionally target plant defensive proteins (Zhu-Salzman and Zeng, 2015).

Recent microscopic observations indicate that spider mites probably have much lower consumption rates than was hitherto assumed (Bensoussan et al., 2016). On common bean (*Phaseolus vulgaris*), the average duration of a single *T. urticae* feeding event was found to last nearly 14 min (Bensoussan et al., 2016), i.e., considerably longer than the roughly 3 s reported earlier (Liesering, 1960). If feeding events indeed last several minutes instead of seconds, pre-digestive functions of secreted salivary proteins would be conceivable. Likewise, this amount of time could allow effectors to interfere with host defenses in the pierced cell prior to ingestion. Additionally, it may allow effectors or their secondary signals to translocate to neighboring cells or to the apoplast to suppress defenses in plant tissues beyond the attacked cell (**Figure 2B**) (Bensoussan et al., 2016; Rioja et al., 2017). Indeed, there is empirical evidence for defense suppression to occur systemically within leaflets (Alba et al., 2015) and within compound leaves (Sarmento et al., 2011a). However, suppression appears to be a predominantly local event, i.e., largely restricted to the mite’s multicellular feeding patch (Schimmel et al., 2017a,b). Molecular studies at single cell resolution are required to assess the true spatial extent of suppression.

We can only speculate about how mite salivary effector proteins operate inside the host plant. Most likely they interact with plant proteins to modulate their function such that the plant becomes more suitable as food. Numerous of such *in planta* targets have been identified for effectors from diverse microbial phytopathogens and for many of these their mode of action has been characterized as well, providing valuable insights into the molecular mechanisms underlying pathogen virulence (Khan et al., 2018; Xin et al., 2018). The majority of phytopathogen effectors as well as their *in planta* targets appear to be of proteinaceous nature (Khan et al., 2018), but note that this could be due to a methodological bias. Large scale protein-protein interaction assays have revealed that a subset of the effectors deployed by phytopathogens targets and modifies a relatively small but conserved set of plant signaling “hubs,” which represent highly connected nodes within the plant protein network, as each of them (potentially) interacts with dozens of other plant proteins (Mukhtar et al., 2011; Wessling et al., 2014). Examples of effector-targeted hub proteins are: TCP transcription factors, which function at the nexus of plant development and defense (Lopez et al., 2015); subunits of the ubiquitin-proteasome system, which are crucial for protein turnover including during phytohormone signaling (Banfield, 2015); JAZ proteins, which are transcriptional repressors of JA responses (Howe et al., 2018), and; papain-like cysteine proteases, which have diverse functions in immune signaling (Misas-Villamil et al., 2016). Consistent with their role in PTI, mutations in effector-targeted hub proteins generally have dramatic consequences for plant resistance to phytopathogens (Mukhtar et al., 2011; Wessling et al., 2014). Other components of the PTI machinery, i.e., that are (relatively) less well-connected, are manipulated by phytopathogen effectors as well. These include conserved detection and signaling components (e.g., PRRs, co-receptors, receptor-like cytoplasmic kinases, MAP kinases, transcriptional regulators; Macho and Zipfel, 2015; Khan et al., 2018) as well as proteins that are produced by the plant to actually fight off the pathogen (e.g., proteases and protease inhibitors; Jashni et al., 2015). Finally, phytopathogen effectors have been found to target proteins with less obvious connections to plant immunity but whose manipulation is nevertheless essential for virulence and pathogen proliferation (Van Schie and Takken, 2014; Macho, 2016). Examples of such so-called susceptibility proteins are: nutrient transporters (Chen et al., 2010), proteins involved in vesicular trafficking (Xin et al., 2016) and cell cycle regulators (Wildermuth et al., 2017). The first reports of proteinaceous plant targets of effectors from herbivorous arthropods indicate that at least some members of this diverse group of plant-feeders may have evolved mechanisms to manipulate their host that are similar to those of microbial phytopathogens. That is, effectors secreted by larvae of the Hessian fly (*Mayetiola destructor*) were shown to interact with the wheat (*Triticum* spp.) Skp subunit of the ubiquitin-proteasome system (Zhao et al., 2015) and effector Mp1 secreted by the aphid *Myzus persicae* was found to interact with Arabidopsis (*Arabidopsis thaliana*) as well as potato (*Solanum tuberosum*) VPS52, which is thought to be involved in vesicular trafficking (Rodriguez et al., 2017). Since mites are not highly mobile, small, and feed from one cell at a time, we hypothesize that there will be considerable overlap between the effector targets of biotrophic microbial pathogens and those of mites, in particular for generalists like *T. urticae*. Specifically, we predict effectors of generalist mites to target conserved plant targets. If so, this would allow the mite to manipulate different hosts using a relatively small set of effectors as compared to the large number of metabolic resistance-conferring genes that would be needed to overcome the defenses of all its different hosts. Specialized mite species may have evolved effectors more specific for their host and as a consequence they may have lost redundant effector paralogs.

Despite the convincing genomic, transcriptomic and proteomic data on mite salivary proteins, the far majority still awaits functional characterization (as effectors). Four mite proteins have been identified as plant defense-suppressing effectors so far; Tu28 and Tu84 from *T. urticae* and the orthologous Te28 (66% identical) and Te84 (63% identical), respectively, from *T. evansi* (Villarroel et al., 2016; Schimmel et al., 2017a). As indicated by their numbers, these proteins represent members from two putative effector families: in *T. urticae* family 28 has 10 members (paralogs), whereas *T. evansi* has only one; family 84 has two paralogs in both mite species (Villarroel et al., 2016). Proteins from these families have also been recovered from the saliva of *T. urticae* feeding on artificial diet (Jonckheere et al., 2016). When transiently overexpressed in *Nicotiana benthamiana* these effectors suppressed SA-defenses (Villarroel et al., 2016) as well as JA-defenses (Schimmel et al., 2017a) and three of the four homologs promoted the fitness of non-adapted *T. urticae* (Villarroel et al., 2016). Te28 did not enhance the performance of *T. urticae* on *N. benthamiana*, probably due to the severe chlorosis that coincided with *Te28* overexpression (Villarroel et al., 2016). In line with this reasoning, on tomato, *Te28* transcript abundance in *T. evansi* correlated negatively with the magnitude of JA and SA defenses in the plant, and positively with mite performance (Schimmel et al., 2017a). Similar correlations were found for *Te84* (Schimmel et al., 2017a), strongly suggesting that Te28 and Te84 are indeed used by *T. evansi* to suppress tomato defenses. The fact that defense-inducing *T. urticae* possess gene copies that encode the functional effectors Tu28 and Tu84 suggests that these mites, too, can suppress defenses. Expression analysis of the corresponding effector genes, though, revealed stunning quantitative differences between *T. urticae* and *T. evansi*, especially for effectors of family 84. On tomato, across different -but comparable- infestation conditions, the relative expression of *Tu28* versus *Te28* ranged from similar levels in the two species to *Te28* transcripts being up to six times more abundant in *T. evansi* than *Tu28* transcripts in non-adapted *T. urticae* (Schimmel et al., 2017a). Transcripts of effector 84 were much more abundant in *T. evansi* regardless of infestation conditions, i.e., *Te84* was roughly 60 to 140 times higher expressed than *Tu84* (Schimmel et al., 2017a). Thus, in addition to differences in the amino acid sequences between orthologous effectors of non-adapted *T. urticae* and specialist *T. evansi*, there are probably also differences at the effector abundance level. Something similar was observed for the spider mite-specific SHOT gene family, which is thought to encode effector proteins (Jonckheere et al., 2017). The genome of generalist *T. urticae* contains 12 SHOT paralogs while Solanaceae-specialist *T. evansi* possess only one ortholog and the Fabaceae-specialist *Tetranychus lintearius* only two (Jonckheere et al., 2017). The expression of several *T. urticae* SHOT genes appeared strongly host-dependent and remarkably plastic, as they were both rapidly and massively induced upon transfer to Fabaceae hosts but were not expressed on plants from other families (Jonckheere et al., 2016, 2017). Together this underscores that the ability of mites to suppress plant defenses via secreted effectors, and possibly to dodge detection by plants, may be tremendously plastic and cannot simply be inferred from the absence/presence of (putative) effectors in the mite’s genome.

As indicated before, there is no information yet on the *in planta* targets of spider mite effectors but based on our knowledge of effectors from microbial phytopathogens (Mukhtar et al., 2011; Wessling et al., 2014; Khan et al., 2018), we speculate that: (a) a subset of the mite effectors will target and manipulate conserved plant proteins that function as signaling hubs in defense and/or development; (b) multiple mite effectors will be able to interact with the same plant protein, while simultaneously; (c) individual mite effectors will be able to interact with multiple plant proteins. Finally, thus far research has been focused on the identification of mite salivary proteins and their characterization as effectors, but effectors are not necessarily of proteinaceous nature. For example, certain bacterial phytopathogens secrete metabolites that function as plant hormones and exploit the conserved hormonal crosstalk mechanism of the host to trigger susceptibility (Zheng et al., 2012; McClerklin et al., 2018). Some eriophyid mites have been suggested to produce and secrete functional plant hormones (De Lillo and Monfreda, 2004). There are no indications that spider mites do so (Grbić et al., 2011). As another example, fungal phytopathogens (Weiberg et al., 2013) and parasitic plants (Shahid et al., 2018) can secrete small RNAs that exploit the host’s RNA interference machinery to silence defense-associated genes. Whiteflies have been predicted to do the same, as they also secrete small RNAs into their host (van Kleeff et al., 2016). The involvement of small RNAs in defense suppression by mites cannot be excluded. Taken together, the mite effector repertoire may extend well beyond their salivary proteins.

To counteract effector-triggered susceptibility, plants have evolved sensory molecules (receptors) often referred to as R-genes/proteins that can by-pass the effector’s manipulation. R-genes usually encode intracellular nucleotide-binding leucine-rich-repeat (NLR) proteins or cell surface-localized receptor-like proteins/kinases (RLPs/RLKs) that detect effectors or effector-activity and subsequently restore PTI plus induce additional defenses that altogether render the plant resistant again (Cui et al., 2015; Kourelis and van der Hoorn, 2018; Su et al., 2018). This R-gene mediated process is referred to as effector-triggered immunity (ETI) (**Figure 2C**). Plant genomes typically contain hundreds of NLR- and RLP/RLK-encoding genes that are fast-evolving and belong to expanded families (Jacob et al., 2013; Kourelis and van der Hoorn, 2018; Su et al., 2018). Consequently, most of these sensory proteins appear to be highly specific, meaning distinct variants are present in each plant species, putatively reflecting the effector repertoire of the biotic attackers they are commonly confronted with (Jacob et al., 2013; Kourelis and van der Hoorn, 2018; Su et al., 2018). This implies that the occurrence of effective R-gene mediated resistance can differ greatly across genotypes (varieties) within plant species. As with PTI, ETI has been well established for plants in response to attacks by microbial phytopathogens, while its involvement during interactions with herbivores is still being explored. For instance, only a small fraction of the R-genes that have been implicated in plant resistance to arthropod herbivores has been characterized to date, i.e., *Mi-1.2* in tomato (Milligan et al., 1998; Rossi et al., 1998; Vos et al., 1998), *Vat* in melon (*Cucumis melo*) (Dogimont et al., 2014) and several *Bph* genes in rice (*Oryza sativa*) (Du et al., 2009; Tamura et al., 2014; Wang et al., 2015; Ji H. et al., 2016; Ren et al., 2016; Zhao Y. et al., 2016; Guo et al., 2018). With respect to mites and ETI, spider mite feeding was shown to rapidly affect the expression of large groups of putative RLK-encoding genes in tomato and Arabidopsis (Martel et al., 2015), suggesting these may play an important role in the detection of mite feeding, i.e., as (co-)receptors for DAMPs, HAMPs, or effectors (Couto and Zipfel, 2016; Kourelis and van der Hoorn, 2018). Other than this report, the involvement of ETI in plant–herbivorous mite interactions (**Figure 2C**) remains hypothetical and requires experimental verification.

The strong selective pressures enforced by such plant receptors is reflected in the characteristics of effector-encoding genes of phytophagous organisms: such genes are usually highly abundant in their genomes, are fast-evolving and belong to expanded families (Jiang et al., 2008; Raffaele et al., 2010; Zhao et al., 2015). This appears to be the case for (putative) spider mite effector genes as well (Jonckheere et al., 2016; Villarroel et al., 2016). Under pressure of ETI, plant-parasites have evolved various counter-adaptations to overcome it, including: (a) the acquisition of sequence mutations in ‘betraying effectors’ that do not interfere with their function yet attenuate recognition by NLRs; (b) the loss of ‘betraying effectors’ via gene silencing or gene removal; (c) the gain of novel effectors that serve as decoys for- or that mask ‘betraying effectors’ (Aggarwal et al., 2014; Dong et al., 2014; Huang et al., 2014; Wei et al., 2015; Ji Z. et al., 2016; Zhao C. et al., 2016; Inoue et al., 2017; Ma et al., 2017; Menardo et al., 2017). Not surprisingly, plant-feeding organisms deploy distinct sets of effectors depending on which host species they attack, likely to deal with the specific defenses they encounter (Yoshida et al., 2016; Mathers et al., 2017; Rivera-Vega et al., 2017; Lorrain et al., 2018). The available data for spider mites is consistent with this hypothesis (Jonckheere et al., 2016, 2017).

## Eco-Evolutionary Background of Plant Defense Suppression by Mites

Plants and herbivores are probably regularly engaged in a co-evolutionary arm’s race. If so, there should be heritable variation in traits that allow plants to resist herbivores as well as heritable variation in traits that allow herbivores to cope with these defenses, for natural selection to act on (Bolnick et al., 2011; Gloss et al., 2016). For interactions between generalists and multiple host plants such interactions are predicted to be more diffuse than for specialists (Futuyma and Agrawal, 2009). Given the tremendous diversity among the more than 200,000 defensive metabolites/proteins found across the plant kingdom, it is hypothesized that the larger the host range of a herbivore is, the smaller is the chance it will evolve metabolic resistance-conferring traits (Becerra, 1997; Despres et al., 2007; Ali and Agrawal, 2012). There are two main arguments to support this hypothesis: (1) Mechanistically, metabolic adaptations to each individual class of defensive metabolites/proteins encountered on diverse hosts do not seem feasible or seem too costly. (2) By changing host species the selective pressure required to evolve and/or maintain metabolic adaptations to specific plant defensive compounds will decrease or disappear. Hence, metabolic resistance-conferring traits are most often found in specialized herbivores, as these feed from a single or a few closely related plants and, thus, continuously encounter the same defensive compounds. By contrast, generalists are hypothesized to increase their fitness across multiple plant taxa by actively interfering with conserved defense signaling components (Ali and Agrawal, 2012; Kant et al., 2015). Concurrently, plant-produced defensive metabolites/proteins are expected to have a different impact on generalist versus specialist herbivores. Whereas generalists are negatively affected at an intermediate level by any class of defensive compounds, specialists are less affected by metabolites/proteins produced by the plant species they have specialized on, but on average suffer more from those produced by non-host plants (Ali and Agrawal, 2012; Heckel, 2014). Studies on various plant-insect systems have found empirical evidence to support these hypotheses (Ali and Agrawal, 2012; Kant et al., 2015), but the available data on plant-mite interactions does not seem to do this for several reasons.

Firstly, among the mites species that have been found to suppress plant defenses, only *T. urticae* is a true generalist, whereas *T. ludeni*, *T. evansi*, and *A. lycopersici* are all (relatively) specialized herbivores, i.e., on Solanaceae (Helle and Sabelis, 1985; Lindquist et al., 1996). Additionally, within natural populations of *T. urticae* the defense-suppressors do not appear to be the dominant genotype (**Figure 1C**) (Kant et al., 2008; Alba et al., 2015). So far, all sampled populations of *T. evansi*, covering both haplotypes, were found to be potent suppressors of tomato defenses (Sarmento et al., 2011a; Alba et al., 2015), suggesting that the defense suppression trait is fixed in this species. These observations apparently contradict the hypothesis that most defense-suppressing herbivores should be generalists. It is worth pointing out, though, that defense suppression by *T. ludeni*, *T. evansi*, and *A. lycopersici*, respectively, has only been demonstrated on cultivated tomato (Sarmento et al., 2011a; Glas et al., 2014; Alba et al., 2015; Godinho et al., 2016) and that, although these mites predominantly infest Solanaceae, they have been found on plants belonging to other families as well. Specifically, *T. ludeni* has been recorded on plants from as many as 62 other families, *T. evansi* on plants from 35 other families, and *A. lycopersici* on one other family, i.e., the Convolvulaceae (Helle and Sabelis, 1985; Lindquist et al., 1996; Migeon et al., 2010). The extent to which *T. evansi* and *T. ludeni* feed from- and reproduce on these non-solanaceous plants is not known. The identification of these mites on non-solanaceous hosts might actually be incorrect (i.e., many *Tetranychus* spp. are hard to distinguish by eye) or be an incidental consequence of passive dispersal (i.e., mediated by wind) from nearby overexploited solanaceous plants (Navajas et al., 2013). Nonetheless, it would be exciting to find out if these (relatively) specialized mites are able to also suppress defenses of plants that do not belong to the Solanaceae.

Secondly, *T. urticae* mites collected from diverse hosts have frequently been shown to be able to adapt to novel hosts. Strangely, most often this adaptation does not seem to go at the expense of their fitness on the ancestral or other hosts (Gould, 1979; Agrawal, 2000; Magalhaes et al., 2009; Wybouw et al., 2015), suggesting *T. urticae* to be a jack-of-all-trades. A comparative genome analysis has revealed that *T. urticae*’s genome harbors expansions in multiple gene families that have been implicated in xenobiotic metabolism, while such expansions were less dramatic, or not found at all, in the genomes of the specialized mites *T. evansi*, *T. lintearius*, and *A. lycopersici*, which suggests that metabolic resistance is a prominent trait underlying *T. urticae*’s adaptive abilities and enormous host range (Grbić et al., 2011; Van Leeuwen and Dermauw, 2016). Accordingly, (experimental evolution) studies that have analyzed the adaptation mechanism(s) of *T. urticae* to novel, challenging host plants have demonstrated large transcriptional plasticity in the mite’s xenobiotic metabolism machinery (Dermauw et al., 2013; Zhurov et al., 2014; Wybouw et al., 2015). Very similar findings have been reported for generalist versus specialist aphids (Ramsey et al., 2010; Silva et al., 2012; Bansal et al., 2014; Mathers et al., 2017; Wenger et al., 2017). However, *T. urticae*’s adaptation to a novel, challenging host plant was also associated with the partial attenuation of a set of plant defense-associated transcriptomic responses, indicative of defense suppression (Wybouw et al., 2015). Something similar was observed for the generalist Kanzawa mite, *Tetranychus kanzawai*, by Ozawa et al. (2017). This suggests that the plasticity in the mite’s effector repertoire (Jonckheere et al., 2016, 2017; Schimmel et al., 2017a) may augment the plasticity in its xenobiotic metabolism to rapidly overcome the resistances of novel hosts. Such a dual mechanism has also been suggested for aphids, whose ability to colonize novel host plants is correlated with transcriptional plasticity of a conserved set of genes, several of which encode (putative) host plant-specific effectors (Elzinga et al., 2014; Thorpe et al., 2016; Eyres et al., 2017; Mathers et al., 2017; Rodriguez et al., 2017). Collectively, the available data suggest that mite traits enabling an improved xenobiotic metabolism are functionally linked, at least partially, with traits related to host defense manipulation.

*Tetranychus urticae* appears to harbor distinct intraspecific variation for traits that cause these mites to induce defenses as well traits that allow them to suppress or to resist tomato JA defenses (Kant et al., 2008; Alba et al., 2015). Both Kant et al. (2008) and Alba et al. (2015) sampled natural populations of *T. urticae* from various non-solanaceous host plants. Subsequently they created near-isogenic lines from individual mites, which were then submitted to a novel host, i.e., WT tomato plants, *def-1* and PS, as described earlier. These assays revealed the existence of three distinct phenotypes (**Figure 1C**): (1) Mites that induce defense responses to which they are also susceptible (i.e., these lower their fitness). This was the most common phenotype. (2) Mites that induced defense responses to which they are resistant (i.e., absence/presence of defense did not affect their fitness). This was a rare phenotype, not found in all populations. (3) Mites that were susceptible to induced defenses but nevertheless had a high performance because they could suppress these defenses. This phenotype was found at low frequencies in all populations. These results suggest that especially the defense-suppression traits could be maintained as a polymorphism by frequency-dependent selection in populations of *T. urticae* living on a mosaic of plant environments. This supports the scenario that defense suppression is a generalist trait that allows it to behave as a jack-of-all-trades, provided that the traits that allow mites to suppress defenses are effective on unrelated hosts. This would be possible if effectors target proteins or processes conserved across multiple host taxa. Yet, since suppression of defenses may come at high ecological costs (Sarmento et al., 2011b; Glas et al., 2014; Alba et al., 2015; Ataide et al., 2016) it may in time be replaced -via natural selection- by resistance traits, which not only appear to be more ‘safe’ in an ecological context, but may also promote fitness stronger than suppression does (Kant et al., 2008). In this scenario, defense suppression will allow populations that shift their host plant frequently to act as jack-of-all-trades but master-of-none. Sub-populations confined to a single host may gain resistance to that host at the expense of suppression and become a master-of-some (i.e., specialized).

Although this scenario predicts that suppression will be rare among specialist this does not seem to be the case for mites, as indicated earlier. This justifies the question why the suppression-traits of mites have not been replaced by resistance-traits during the course of specialization? We argue that these species possess buffering traits that can shield suppression-traits from natural selection imposed by facilitated competitors and/or natural enemies.

## Buffering Traits That Enable Mites to Mitigate Negative Side Effects of Host Defense Suppression in Natural Communities

**FIGURE 3:**
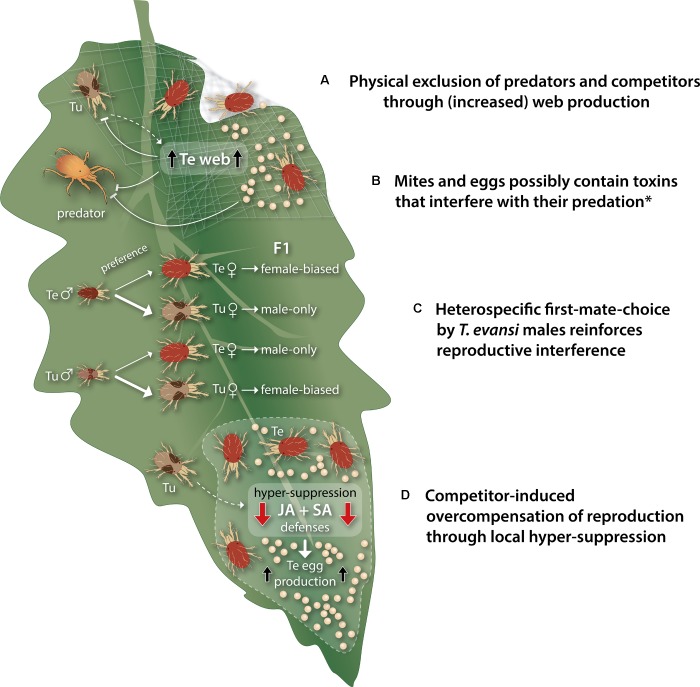
Schematic, simplified overview of the buffering traits that may enable *Tetranychus evansi* mites to mitigate negative side effects of host defense suppression that can occur when living in natural communities. **(A)** *T. evansi* (Te) covers its feeding site with copious amounts of silken web, which shields off competing *Tetranychus urticae* (Tu) as well as predatory mites. Moreover, upon perception of *T. urticae* cues (dashed arrow), *T. evansi* produces a denser web, presumably to increase exclusion of its competitor. **(B)** Multiple species of predatory mites have an extremely poor performance on a *T. evansi* diet, possibly because *T. evansi* mites and their eggs contain one or more host plant-derived toxins that interfere with their predation. **(C)** Contrary to *T. urticae* males, *T. evansi* males prefer to copulate with heterospecific (i.e., *T. urticae*) females, this asymmetric mating preference reinforces reproductive interference by *T. evansi*, which can negatively impact the population growth of competing *T. urticae*. Spider mites are haplodiploid organisms and females show a strong first-male sperm precedence. Heterospecific mating events, therefore, result in (near) male-only offspring (hybrid females are not fertile), whereas conspecific mating events yield strongly female-biased offspring. **(D)** Upon perception of cues from nearby *T. urticae* (dashed arrow), *T. evansi* mites hyper-suppress jasmonate (JA) and salicylate (SA)-regulated plant defenses, albeit only at their feeding site, and this is paralleled by an increased oviposition rate for *T. evansi*, while the invading *T. urticae* does not benefit (yet). This rapid overcompensation response likely boosts *T. evansi*’s competitive population growth. The asterisk denotes that there is currently no empirical evidence for a causal relationship between the sequestration of toxins by *T. evansi* and the increased predation risk that may be associated with defense suppression by these mites.

Probably the most obvious of such buffering traits of *T. evansi* concerns the production of web. As a family characteristic, spider mites produce silk, which is among others used to construct a web that shields the mites from unfavorable abiotic conditions as well as from competitors and predators (Helle and Sabelis, 1985). Silk production quantitatively differs between spider mite species and *T. evansi* is known to synthesize extraordinarily large amounts of it (Helle and Sabelis, 1985). Shortly after colonization of a new host plant, when the population size is small, only local feeding patches are covered with web, but as the population grows, entire plants get readily encapsulated (Liu et al., 2017). The particularly dense web of *T. evansi* effectively hinders competing herbivorous mites, such as *T. urticae* (Sarmento et al., 2011b), as well as predatory mites, like *Euseius concordis* (De Moraes and Lima, 1983) (**Figure 3A**), but there is more to it than that. Results from another study suggest that *T. evansi* may actively increase the exclusion of competitors, as *T. evansi* females were found to produce a denser web in response to cues emanating from nearby *T. urticae* feeding sites (Sarmento et al., 2011b) (**Figure 3A**). The same happened in response to local *T. urticae* cues (Sarmento et al., 2011b). Vice versa, *T. evansi* does not appear to be hindered by *T. urticae*’s web, nor does *T. urticae* produce a denser web when *T. evansi* feeds close by (Sarmento et al., 2011b). Surprisingly, local cues from the predatory mite *P. longipes* did not trigger an increased production of web by *T. evansi* (Lemos et al., 2010). Different predator-induced behavioral changes were observed instead: not only did *T. evansi* lay fewer eggs, about a third of its eggs were suspended in the web, whereas nearly all eggs were deposited on the leaf surface under predator-free conditions (Lemos et al., 2010). Compared to eggs on the leaf surface, web-suspended eggs were less likely to be eaten by *P. longipes* (Lemos et al., 2010), providing a clear explanation for *T. evansi*’s altered behavior.

Predation of *T. evansi* eggs is actually a relatively rare event in nature, especially outside *T. evansi*’s native area (Navajas et al., 2013). For numerous naturally co-occurring as well as commercially available predatory mites, *T. evansi* is an unsuitable prey, meaning that aside from the adverse effects of the silken web and host-plant trichomes, most predators have an extremely poor performance on a diet of *T. evansi*, likely because it is toxic (De Moraes and Mcmurtry, 1985; Escudero and Ferragut, 2005; Rosa et al., 2005; Ferrero et al., 2014). This toxicity has been attributed to one or more plant-derived metabolites, which are probably modified and/or sequestered by *T. evansi* and passed on to their eggs as well (**Figure 3B**) (De Moraes and McMurtry, 1986; Koller et al., 2007; Ferrero et al., 2014). Selection for this toxin sequestration has possibly been promoted by an increased predation risk due to suppression of defenses as suggested by Ataide et al. (2016). It should be noted that defense suppression by *T. evansi* does not necessarily prevent the attraction of predatory mites, i.e., indirect plant defenses, despite their interference with the herbivory-induced production of volatile organic compounds (Sarmento et al., 2011a; Lemos, 2015). Hence, the toxin sequestration may be a buffering trait.

The third buffering trait of *T. evansi* concerns the direct interference with *T.* *urticae*’s reproduction due to asymmetric mating preferences. Even though the two species are reproductively incompatible, *T. evansi* males prefer to mate with *T. urticae* females instead of with conspecific females, whereas *T. urticae* males *do* preferentially mate with conspecifics (Sato et al., 2014, 2016). Since spider mites are haplodiploid organisms and females show a strong first-male sperm precedence (Helle and Sabelis, 1985), this asymmetric mating preference can have a strong negative effect on *T. urticae*’s population growth when mites from both species co-occur (Sato et al., 2014), a phenomenon known as reproductive interference (**Figure 3C**). That is because although heterospecific mating events do not affect the total number of eggs laid, females produce predominantly male offspring upon mating with a heterospecific male, as opposed to strongly female-biased offspring when fertilized by a conspecific (Sato et al., 2014; Clemente et al., 2016). The few hybrid females derived from interspecific mating events between *T. urticae* and *T. evansi* are not fertile (Clemente et al., 2016). Reproductive interference has also been observed between *T. urticae* and *T. ludeni* (Clemente et al., 2017), but it is not known which effects this has on the population growth of both species.

The fourth buffering trait of *T. evansi* involves plasticity in its reproductive performance -possibly resulting from plasticity in the magnitude of suppression- in response to the presence of competitors (**Figure 3D**). Analogous to the competitor-induced increased web production, *T. evansi* females on a well-established feeding site were found to suppress plant defenses stronger, albeit only locally, when *T. urticae* was introduced to adjacent leaf tissue (Schimmel et al., 2017a). This local hyper-suppression coincided with the increased expression of effector-encoding genes in *T. evansi* (*Te28* and *Te84*) and, moreover, was paralleled by an increased production of eggs by *T. evansi* -but not by the invading *T. urticae* (Schimmel et al., 2017a). Also Orsucci et al. (2017) found evidence for an increase in *T. evansi*’s reproductive performance when *T. urticae* was present on the same tomato leaf. In the opposite experimental situation, no significant changes were detected in the plant’s defense responses, nor did *T. urticae* females produce more eggs upon introduction of *T. evansi* to adjacent leaf tissue (Schimmel et al., 2017a). This competitor-induced, plant-mediated overcompensation response of *T. evansi* therefore likely promotes its competitive population growth on tomato.

The discovery and characterization of *T. evansi*’s buffering traits has raised numerous questions, in particular whether similar traits can be found in other defense-suppressing mites (or insects)? For *A. lycopersici* the answer is a partial no, because this species does not produce web at all. However, this mite is extremely small and resides exclusively within the trichome forest on tomato stems and leaves, which is neither accessible for *T. urticae* nor for predatory mites. This may represent a behavioral trait that buffers facilitating competitors or natural enemies. Interestingly, after a few days of feeding by *A. lycopersici* glandular- and non-glandular trichomes on tomato deteriorate and this exposes the mite to its natural enemies, such as the predatory mite *Amblydromalus limonicus*. On such plants the russet mites were observed to rapidly move toward plant parts with intact trichomes (Van Houten et al., 2013).

Taken together, although natural selection may act against defense suppression under pressure of competition and predation this trait may also escape selection when shielded by buffering traits. These buffering traits may allow defense suppressors to remain suppressors, i.e., to counteract the evolution of resistance, during periods of specialization by enabling them to maintain the monopoly on their feeding site and to exclude natural enemies.

## Conclusions and Perspectives

So why do herbivorous mites suppress plant defenses?

(1) Not all herbivorous mites seem to suppress plant defenses but those that do obviously benefit from suppression as it increases their performance under laboratory conditions.
(2) Under natural conditions the benefits of suppression are less obvious since the ecological risks (costs) that come with suppression can be considerable.
(3) Suppression of defenses by herbivores is facilitated by secreted salivary effector proteins that manipulate plant processes to turn their host into a better food source.
(4) In our view the ability to suppress defenses facilitates a generalist life style and for these generalists -that move across environments with variable ecological risks- the advantage of being able to colonize multiple hosts may, on average, outweigh the costs.
(5) Existence of intraspecific variation suggests suppression-traits of generalist herbivores that live on a mosaic of plant environments to be maintained by frequency-dependent selection.
(6) We predict the effectors of generalists to target elements (e.g., proteins) of plant processes (e.g., defense pathways), that are conserved across their multiple hosts and thereby facilitate their multiple-host life style. This in contrast to xenobiotic resistance that will usually only facilitate a herbivore’s compatibility with a limited set of (related) plant hosts.
(7) Evidence suggests that upon colonization of a novel host by the generalist *T. urticae* the ability to suppress defenses rapidly emerges possibly due to plasticity and/or selection.
(8) We predict that in generalists confined to a host for extended periods of time the suppression trait will be replaced by resistance traits, because these traits are ecologically more safe and, according to the data available, may promote mite performance more strongly.
(9) We argue that the existence of specialists that suppress defenses rather than resist them may represent ‘accidents’ facilitated by buffering traits that shield suppression from natural selection. We predict these specialists to possess a smaller set of effectors/effector paralogs than generalists do and these to more often target less conserved (i.e., more host-specific) plant proteins or processes.
(10) We speculate that under the umbrella of the buffering traits, the suppression traits of specialists may still erode because of physiological costs and/or drift, yet at a relatively slow pace.
(11) Finally, we argue that defense suppression traits and their buffering traits can be, but not necessarily are, co-adaptations.

## Author Contributions

MK and BS conceptualized the manuscript. CB, EV-P, and RC drafted the manuscript. TVL, MK, and BS supervised the writing, critically revised the manuscript, and contributed to its final version. CB, EV-P, RC, and BS designed the figures.

## Conflict of Interest Statement

The authors declare that the research was conducted in the absence of any commercial or financial relationships that could be construed as a potential conflict of interest.
